# Editorial: Omics data-based identification of plant specialized metabolic genes

**DOI:** 10.3389/fpls.2023.1209334

**Published:** 2023-06-09

**Authors:** Peipei Wang, Pengxiang Fan, Yan Bao, Wei Li, Li Wang

**Affiliations:** ^1^Traditional Chinese Medicine & Floriculture Research Center, Kunpeng Institute of Modern Agriculture at Foshan, Foshan, Guangdong, China; ^2^Agricultural Genomics Institute at Shenzhen, Chinese Academy of Agricultural Sciences, Shenzhen, Guangdong, China; ^3^Department of Horticulture, Zhejiang University, Hangzhou, China; ^4^Shanghai Collaborative Innovation Center of Agri-Seeds, Joint Center for Single Cell Biology, School of Agriculture and Biology, Shanghai Jiao Tong University, Shanghai, China

**Keywords:** specialized metabolic genes, metabolome, transcriptome, plant natural products, evolutionary history

Plant natural products, especially specialized metabolites, are major sources of nutrients, medicines and industrial materials. Identifying genes responsible for the biosynthesis of plant natural products has crucial significance but also has always been a recognized challenge in synthetic biology, due to the lineage-specific distribution and the fast evolution of plant natural products, the complex evolutionary history of underlying genes, the interweaving network of metabolic pathways, and the time-consuming and laborious experient processes, etc. In this Research Topic, 97 authors contributed 12 original research and review manuscripts, primarily focused on the identification of plant specialized metabolic genes via integrating multiple omics data.

Transcriptome and metabolome are dynamic and closely related. Comparing and integrating these two omics data has been widely used to identify key genes responsible for the biosysthesis of specialized metabolites. Phthalides from roots of the medicinal herb *Angelica sinensis* are the main chemical components for promoting blood circulation. By integrating metabolome and transcriptome from roots of two groups of *A. sinensis*, Feng et al. proposed the reaction pathway for phthalide biosynthesis, identified and validated six enzyme genes involved in this biosynthesis pathway. Li et al. used a similar strategy to compare volatile metabolome and transcriptome between two varieties of *Lonicera japonica* and gained insights of floral scents regulation in these two varieties. Yang et al. examined the different cumulation of benzylisoquinoline from different tissues of *Sinomenium acutum*, a medicinal plant used for treating rheumatoid arthritis for hundreds of years, and identified candidate genes responsible for benzylisoquinoline alkaloid biosynthesis via integrating the metabolome and full-length transcriptome data. Wang et al. selected two varieties of the high-value nut crop, *Macadamia integrifolia*, each exhibiting distinct floral traits, such as flower coloration and aroma formation. They revealed a metabolic network integrating genes associated with hormone signal transduction, starch and phenylpropanoid metabolism, which play roles in the development of flower coloration and aroma.

Aiming to reveal the formation mechanism of red stem in *Prunus mume*, a graceful horticultural plant known for its varied colors and postures, Qiu et al. identified metabolites in anthocyanin-related pathways, particularly cyanidin glycoside and paeoniflorin glycoside, that are only accumutated in the “Wuyuyu” accession with red stem rather than in the “Fei Lve” accession with green stem. They also identified several genes which are potentially involved in anthocyanin metabolic pathways for the red pigment formation using transcriptome data. Liu et al. identified several candidate genes for the biosynthesis of gastrodin, a main bioactive ingredient of a medicinal plant *Pholidota chinensis* Lindl., which is used to treat high blood pressure, dizziness and headache.

Lotus plumule is a green tissue in the middle of seeds that predominantly accumulates bisbenzylisoquinoline alkaloids and chlorophyll. Sun et al. identified potential enzyme genes responsible for bisbenzylisoquinoline alkaloids and chlorophyll biosynthesis in Lotus plumule by comparing time series profiles of these two types of metabolites and the transcriptome in this tissue. By conducting gene family evolution analysis and examining gene expression pattern in different tissues and under UV-B treatment, Ren et al. identified two flavonoid glucosyltransferases responsible for flavonoid glucosylation in Chinese bayberry *Mor*ella *rubra*. To elucidate the metabolic and transcriptomic basis for toon bud deterioration after harvest, Zhao et al. explored the metabolic regulation of *Toona sinensis* toon buds in different postharvest storage temperatures via metabolic profiling and gene expression analysis, and identified several metabolic pathways whose changes after harvest might contribute to the toon bud deterioration.

Tocopherols are widely recognized as vitamin E, which is an essential nutrient in the human diet. As a lipid-soluble antioxidant, vitamin E plays a broad and fundamental role in controlling seed longevity and viability, high-light acclimation, and cold response, among other functions. In a recent study, Niu et al. summarized recent progresses in the biosynthesis and response of vitamin E. Key discoveries in recent years include the identification of the seed-specific α/β hydrolase VTE7 for tocopherol biosynthesis, the genetic connection between vitamin E metabolism and miRNA biogenesis, and the critical role of vitamin E in mediating plant cold response. This review provides valuable information for tracking the progresses of vitamin E synthesis and signaling pathways.

The exploration of evolutionary history of genes involved in the secondary metabolite biosynthetic pathways can provide insights into the biological processes in land plants. At the macro-evolutionary scale, Kruse et al. investigated the patterns of sequence and expression evolution of BAHD acyltransferase family in land plants. They found that the gene family expansions are concordant with the prominence of metabolite classses and most co-expressed BAHDs in rice and *Arabidopsis* belong to distinct clades. At the micro-evolutionary scale, Marszalek-Zenczak et al. unveiled the evolution of four metabolic gene clusters (MGCs) in *Arabidopsis thaliana* populations covering around 1,000 accessions. Marneral and tirucalladienol MGCs are rather conserved, while thalianol and especially arabidiol/baruol MGCs display profound diversity among accessions. The arabidiol/baruol MGC contained divergent duplicates of both *CYP705A2* and *BARS1* genes in one-third of accessions, which was correlated with the root growth dynamics and adaptation to climate changes. These two studies indicate that the evolutionary history of metabolic genes sheds light on the gene functional prediction and phenotypic diversity of plants.

In summary, this Research Topic features diverse research efforts and methodologies aimed at identifying plant specialized metabolic genes, through integrating and comparing multiple omics data types. These studies have uncovered key genes involved in specialized metabolite biosynthesis across a wide variety of plants, providing insights into the molecular mechanisms that underlie the production of valuable plant-derived compounds. The knowledge gained from this Research Topic has the potential to pave the way for enhancing production and utilization of these valuable compounds in various applications, spanning the nutritional, pharmaceutical, and ornamental industries.

## Author contributions

All authors listed have made a substantial, direct, and intellectual contribution to the work and approved it for publication.

